# Technology, Training, and Task Shifting at the World’s Largest Mass Gathering in 2025: An Opportunity for Antibiotic Stewardship in India

**DOI:** 10.2196/45121

**Published:** 2023-03-08

**Authors:** Isaac H Y Chan, Miriam Gofine, Shitij Arora, Ahmed Shaikh, Satchit Balsari

**Affiliations:** 1 Lakshmi Mittal and Family South Asia Institute Harvard University Cambridge, MA United States; 2 Department of Population Health NYU Grossman School of Medicine New York, NY United States; 3 Vilcek Institute of Graduate Biomedical Sciences NYU Langone Health New York, NY United States; 4 Division of Hospital Medicine Department of Internal Medicine Montefiore Medical Center New York, NY United States; 5 Institute for Critical Care Medicine Mount Sinai Hospital New York, NY United States; 6 Department of Emergency Medicine Beth Israel Deaconess Medical Center Harvard Medical School Boston, MA United States

**Keywords:** digital tools, mass gathering, Kumbh Mela, antibiotics, antimicrobial, stewardship, surveillance, public health, informatics, India

## Abstract

The role of antibiotic overuse in intensifying selection pressures and contributing to the emergence of antimicrobial resistance is well established. The Kumbh Mela, a religious festival that occurs in 4 Indian cities of spiritual significance, is the world’s largest mass gathering, attracting over 80 million pilgrims in 2013. Digital syndromic surveillance from the 2013 and 2015 Melas demonstrated a consistent pattern of antibiotic overuse, with an antibiotic prescribing rate of up to 31% for all patient encounters. As preparations for the 2025 Kumbh Mela begin, task shifting, point-of-care diagnostic and digital tools, robust clinician training, and community awareness can promote the restrained and evidence-based use of antibiotics, minimizing the potential for the emergence of antimicrobial resistance at the world’s largest mass gathering.

## Introduction

The World Health Organization’s global action plan on antimicrobial resistance (AMR) recognizes that AMR “threatens the very core of modern medicine and the sustainability of an effective, global public health response to the enduring threat of infectious diseases” [1]. The contribution of antimicrobial use and overuse in intensifying selection pressures that lead to the emergence of AMR is well established [2]. India has the highest rate of human antibiotic use in the world and has a substantial burden of antimicrobial-resistant organisms as well [3]. Both the overuse of antimicrobial agents and the emergence of resistant organisms are exacerbated during stressors to population health, as seen during the COVID-19 pandemic [4-6]. These phenomena are also seen in certain regularly scheduled events like large mass gatherings where millions congregate over several days and where wanton prescription of antibiotics has been observed [7].

The Kumbh Mela is one such religious festival held periodically in 4 cities of spiritual significance in India [8]. Millions from across the country visit the weeks-long festival for a religious “dip” in the holy river waters on the banks of which the Melas are held. The Melas were last held in Allahabad in 2013, in Nashik in 2015, in Ujjain in 2016, and in Haridwar in 2021. Our digital surveillance at 2 of these events in the past, including at the 2013 Kumbh Mela (the world’s largest mass gathering to date), shows that antibiotic stewardship at the publicly funded health care facilities at these events is nearly nonexistent [9,10].

Planning for the Melas begins nearly 1 to 2 years in advance, presenting a unique opportunity to strengthen public health systems and improve antibiotic stewardship in time for the 2025 Kumbh Mela in Allahabad, which is again expected to set the record for the largest mass gathering in history. In this viewpoint, we argue for the restrained use of antibiotics; the use of digital tools to monitor, nudge, and support evidence-based prescribing; and for robust clinician training and community awareness to modify expectations and change practice in anticipation of, and during, the 2025 Kumbh Mela.

## The 2013 and 2015 Kumbh Melas

Clinical encounters in the 2013 and 2015 Melas in Allahabad and Nashik, respectively, were digitally documented via a syndromic surveillance system deployed at both Melas [8-10]. Some of the authors (SB and AS) were directly involved in extensively studying various aspects of public health preparedness at these Melas and were responsible for the design and implementation of the digital systems, the first of cloud-based syndromic surveillance systems deployed at scale. It is now well documented that significant resources are directed toward public health preparedness and public safety at the Melas, in response to cholera outbreaks and stampedes that have marked previous iterations of the gathering [11]. At each festival, the state and central governments cooperate to provide a range of services for the visiting pilgrims, including temporary shelters, free meals, subsidized transport, and health care. Temporary health clinics are staffed by physicians seconded from other government-run primary clinics across the host state. The clinics are characterized by very high patient volumes, where each provider may see hundreds of patients a day. The doctor-patient encounter is cursory, and patients expect to be prescribed medications for their ailments, in concordance with known health-seeking behavior in India [12,13]. Protocols established by the state health ministry allow the provision of only a 3-day supply of antibiotics, recommending a follow-up visit [9]. With some exceptions, the vast majority of pilgrims only make a day trip to the Mela and return home.

The 2013 syndromic surveillance system was deployed at the 4 busiest of 13 clinics at the Allahabad Mela and at the larger secondary care hospital on site [8,9]. The clinics provided free services to the 80 million pilgrims who visited the Mela over a 55-day period. Roughly 280,000 patients were seen in the clinics during the Mela, and data were captured from 49,131 of these encounters [8,9]. We found that as the number of visitors rose on certain auspicious days, clinic visits spiked with some sites seeing over 2000 patients a day [9]. On February 9, the busiest of all days at the 2013 Mela, over 30 million pilgrims visited the festival. The median age in 49,131 patient encounters recorded at the 5 study sites was 46 years, the majority being men (70%) [9]. The most common recorded presenting complaints were musculoskeletal pain (19%), fever (17%), cough (17%), coryza (16%), and diarrhea (5%) [9]. A total of 91% of patients at the 4 sector clinics and nearly 70% at the central hospital received at least 1 or more drug prescriptions. Of all patient encounters, 31% received an antimicrobial agent [9]. Of patients who presented with a complaint of subjective fever (n=8490), nearly half received a 3-day prescription for antibiotics, empirically, without any confirmatory tests [9].

**Figure 1:**
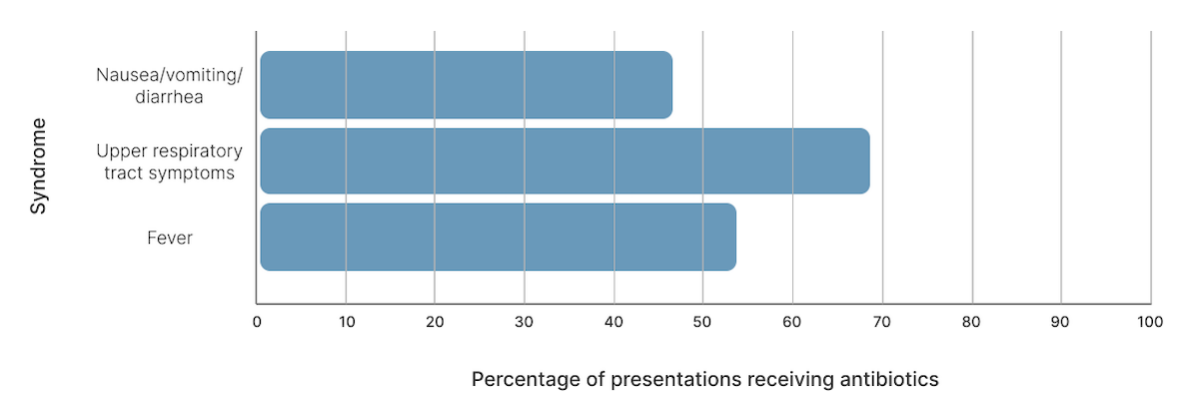
Percentage of patients prescribed antibiotics based on their syndromic presentation.

The syndromic surveillance exercise was repeated 2 years later at the Nashik Kumbh Mela, which was attended by nearly 30 million people [10]. Data were collected from over 40 clinics servicing the visiting pilgrims in the host cities of Nashik and Trimbakeshwar [10]. The clinics mainly ran on the most auspicious days, totaling four 3-day runs of operation, sometimes including both sites. A total of 33,305 unique patient encounters were captured over 9 days. The most common presenting complaints were upper respiratory tract symptoms (n=10,866), joint pain (n=5730), and fever (n=4861). Prescription data were available for 14,725 patient encounters from a subgroup of randomly selected clinics. Within this group, of the 2067 patients who presented with subjective fever, half (n=1111, 53.7%) were prescribed antibiotics empirically (as they were at the 2013 Mela). Among 3921 patients presenting with upper respiratory tract symptoms, 2692 (68.7%) received antibiotics. Of the 1429 patients with gastrointestinal complaints, 668 (46.7%) were prescribed antibiotics (Figure 1).

**Figure 2:**
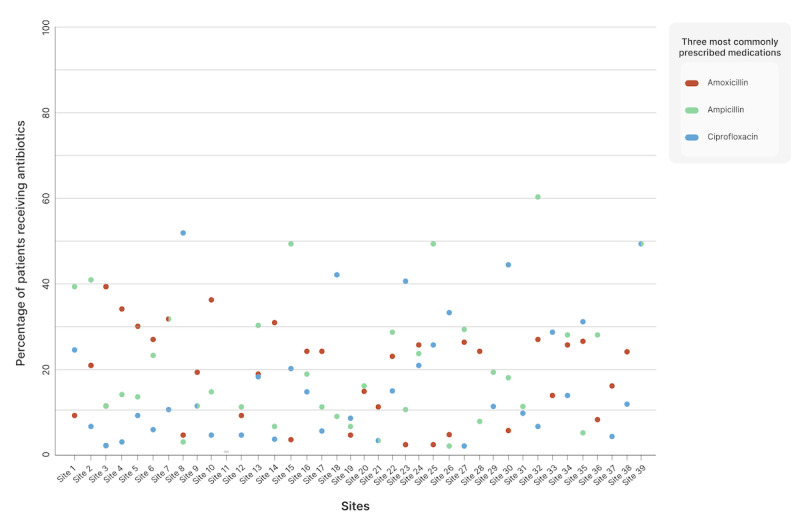
Variation in antibiotic prescription practices across sites for patients presenting with upper respiratory tract symptoms at the 2015 Kumbh Mela.

These findings are concerning for several reasons. Data from these Melas show that antibiotic prescribing rates can be as high as 31% for all encounters. Almost 69% of patients with upper respiratory tract symptoms received antibiotics, an alarmingly high rate, given the overwhelming evidence that the vast majority of upper respiratory tract infections are caused by viral pathogens [14]. These rates are concordant with previously published estimates of antibiotic prescription rates from undifferentiated outpatient settings in India, which range from 39% to 66% [15-17]. There is also substantial interprovider variation in prescribing practices (Figure 2 and Multimedia Appendices 1 and 2). When antibiotics are used, there is little consistency or evidence-based practice to guide decision-making when choosing antibiotics. Across all clinics at the 2015 Kumbh Mela, the rate of fluoroquinolone prescription for patients with upper respiratory tract symptoms ranged from 0% to over 50%, with similar variation in the prescription of other antibiotics. Such use of broad-spectrum antibiotics as empiric therapy is consistent with other observations from India and indicates the need for ongoing education for providers [18,19].

## Policy Recommendations

While well intentioned, the provision of health services for low-acuity illnesses at the Kumbh Mela needs urgent reform. Preparation for the 2025 Allahabad Kumbh Mela commences now as the state assembles its administrative team and various contractors begin to reactivate their supply chains. The Mela administration will have a dedicated team dealing with public health preparedness and health services. Now is the time to reimagine what these should look like in 2025, for what is likely to become the world’s largest mass gathering.

Evidence from the prior 2 Melas we studied has shown the following: the vast majority of presentations are of low acuity; there are little to no diagnostics available except at the district hospital; there is near universal overprescription of antibiotics; and when prescribed, the choice and dosage of antibiotics chosen appears arbitrary. Each of these observations warrants attention as planning for the Kumbh begins.

Our recommendations to promote antimicrobial stewardship at the Kumbh Mela are summarized in Table 1.

**Table 1:** Recommendations to promote antimicrobial stewardship at the Kumbh Mela, the world’s largest mass gathering.

Intervention	Impact on antimicrobial stewardship
Highly protocolized triage by mid-level providers (task sharing)	Clinically stable patients not requiring medications are discharged with symptomatic management and patient education
Point-of-care diagnostics (technology)	Targeted, rapid diagnostics may prevent unwarranted prescriptions of antibiotics or help better target therapy
Physician education (training)	Simple, user-centered clinical protocols can guide appropriate antibiotic prescription
Technological assistance (technology)	Benchmarking of one’s antibiotic prescription rate against peers acts as a behavioral nudge that rationalizes prescribing practice
Patient education (training)	Patient-facing education initiatives address the patient expectations that drive the demand for antibiotics in the Indian context

### 1. Task Sharing

All clinics are staffed by trained allopathic physicians. As epidemiological data from these 70,000 patient encounters have consistently shown, patients at the Mela often present with low-acuity symptoms, many of which likely warrant no diagnostics or therapeutics, and most likely do not need the attention of a physician—especially one that has been drawn away from a busy clinic elsewhere in the state in order to participate in a fleeting patient encounter.

In recent years, the Ministry of Health and Family Welfare has been promoting task sharing with mid-level practitioners and with trained nonallopathic physicians. Given what is known about the epidemiology of patient presentations at the Mela, highly protocolized triage by mid-level providers, community health workers, and senior medical students can identify patients who do not require any intervention, as well as those that require a detailed physician review. Patients who meet predefined criteria for clinical stability can be discharged. Such task sharing allows for a more effective allocation of limited physician resources. Fewer patient encounters per physician may reduce decision fatigue, which also contributes to antibiotic initiation [20].

### 2. Point-of-Care Diagnostics

The proposed task shifting will enable richer doctor-patient interactions for those who have been identified by first-line providers as needing additional attention. Physicians at previous Melas spent less than 3 minutes per patient encounter, often prescribing antibiotics without examining the patient [9]. Previous Melas have also been characterized by a lack of diagnostic capacity, with no laboratory or radiology services outside of the central referral hospital [9]. As a result, only 0.3% of patient encounters at the 2013 Mela used x-rays and almost none received blood tests [9]. The absence or underutilization of diagnostic testing may lead to well-intentioned but inappropriate initiation of empirical antibiotic therapy. Many diagnostic tests, including radiography and rapid antigen testing, for example, can now be performed at the point of care. Such diagnostics must be considered for an appropriately triaged subset of patients in whom empiric therapy cannot be justified.

### 3. Regulating Antibiotic Prescription

Promoting the appropriate use of antibiotics remains a significant challenge, and multiple approaches have been tried around the world. A multipronged approach will be necessary to promote antibiotic stewardship at the Kumbh Mela.

#### Physician Education

Sustained provider education, streamlined clinical decision support systems, standardized treatment algorithms, and behavioral nudges are core components of antimicrobial stewardship programs [21]. The Kumbh Mela, which runs for several weeks, provides unique access to large numbers of physicians visiting from around the state. The education of prescribers involved in antibiotic use is a core pillar of antibiotic stewardship [21]. Training them in simple, user-centered clinical treatment protocols with standardized thresholds for antibiotic initiation in the weeks leading up to the Mela may help streamline clinical decision-making and promote the rational use of antimicrobial agents. Resources permitting, following a cohort of these physicians over time as they return to their hometowns may offer insights into the sustainability of these interventions and their generalizability to antimicrobial stewardship programs in India at large. Of note, the current policy of providing a 3-day supply of antibiotics for ailments diagnosed at the Mela should be reexamined, as it risks both overtreating patients without bacterial infection and undertreating those that do. In the case of pneumonia, for example, society guidelines in India and abroad recommend at least 5 days of antibiotic therapy [22,23].

#### Technological Assistance

**Figure 3:**
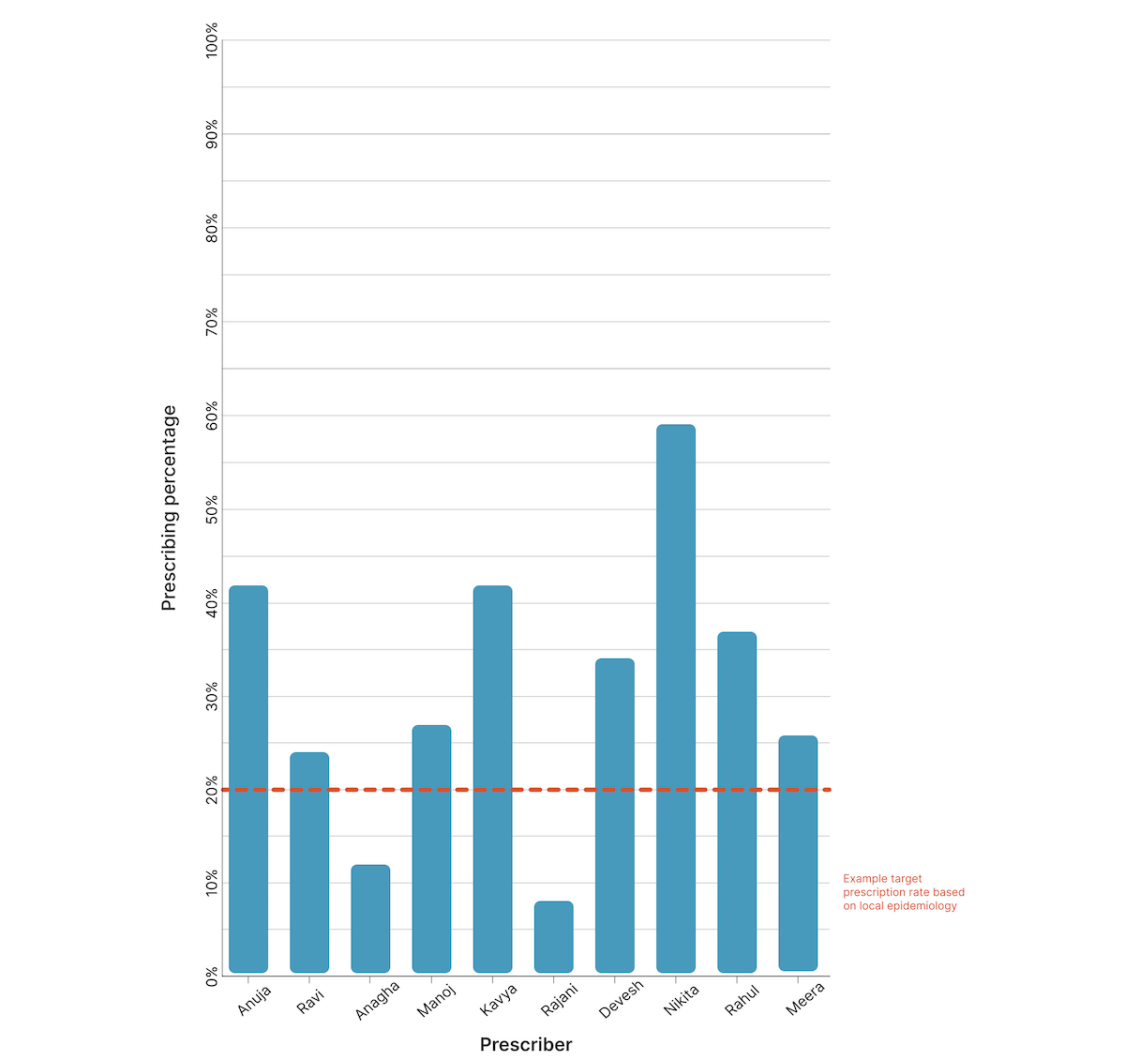
Proposed end-of-the-day feedback to prescribers at the Kumbh Mela demonstrating their prescribing practices compared to their peers and to a validated benchmark of expected antibiotic utilization, according to local epidemiology.

The Mela provides an excellent opportunity to build on prior successes with lightweight, mobile digital health tools to promote sustainable antimicrobial use. An electronic medical record, which has already been successfully deployed at previous Melas in the form of a tablet-based app, will allow for performance scores and quality metrics related to rates of antibiotic prescribing. Anonymized benchmarking of providers against their peers may serve as an effective behavioral nudge to rationalize prescribing practice (Figure 3). The use of electronic prescribing, as envisioned by the Ayushman Bharat Digital Health Mission (ABDHM), and clinical decision support tools embedded within the electronic medical record could further support good prescribing practice [24]. These initiatives can inform broader efforts to regulate antibiotic sales in India, which is characterized by high levels of over-the-counter dispensing of antimicrobial agents [25].

#### Patient Education

A particular challenge in the Indian context is that of patient expectations that drive demand for antibiotics, and risk aversion among providers, leading to inappropriate initiation of empiric antibiotic therapy [26]. The Mela provides an ideal opportunity for initiating a conversation about antibiotic use with the broader public, as has been done with other themes at previous Melas (eg, the 2015 Kumbh Mela was designated as an eco-friendly “Harit Kumbh” [Green Kumbh], with a focus on sustainability [27]). Awareness campaigns at congregation spaces and in the vicinity of clinics are likely to be noticed.

## Limitations and Ethical Considerations

Digital tools may facilitate responsible antibiotic prescribing through education, monitoring, and surveillance, but they must be combined with other core elements of antimicrobial stewardship, including a strong commitment to stewardship by local and national health authorities, as well as ongoing reporting and feedback [21]. The proposed digital tools will work best as part of the local and national commitment to antimicrobial stewardship.

Ethical issues surrounding digital tools and data privacy in health care records must be considered [28]. In India, there has been a robust debate regarding the governance framework for health data exchange in light of the ABDHM [28]. Similar digital tools to the ones proposed above have been deployed at previous iterations of the Mela without undermining individual privacy [8-10]. Peer-based benchmarking of antibiotic prescription rates will require anonymized and aggregated prescribing data to allow for supportive and nonpunitive learning opportunities [28]. Such anonymization is at least theoretically possible within the digital architecture proposed by the ABDHM.

## Future Directions

Despite the scale of the threat posed by AMR, stewardship activities have been difficult to implement not just in India but across the world. India continues to be the world’s largest consumer of antibiotics, but there is limited evidence to guide antimicrobial stewardship activities in the Indian context [3]. The government’s support for task shifting and focus on technology and public angst over the state of health care in the aftermath of the COVID-19 pandemic provide an impetus for change. Given the tens of millions that are expected to converge at the Kumbh Mela in Allahabad in 2025, bold measures to combat antibiotic overuse and AMR are urgently required. The Mela offers a unique opportunity to deploy and validate digital interventions in an environment with multiple stressors and can act as a catalyst for sustained, larger-scale interventions across the Global South. Lessons learned from the Kumbh Mela may also inform antimicrobial stewardship activities at other organized mass gatherings, such as the Hajj in Saudi Arabia, which face similar challenges related to antibiotic overuse [29,30].

## Appendix

Multimedia Appendix 1 Variation in antibiotic prescription practices across sites for patients presenting with self-reported fever at the 2015 Kumbh Mela.

Multimedia Appendix 2 Variation in antibiotic prescription practices across sites for patients presenting with gastrointestinal symptoms at the 2015 Kumbh Mela.

## Abbreviations

ABDHM: Ayushman Bharat Digital Health Mission
AMR: antimicrobial resistance
UNICEF: United Nations Children’s Fund
