# Supplementary figures and images for: Technology, Training, and Task Shifting at the World’s Largest Mass Gathering in 2025: An Opportunity for Antibiotic Stewardship in India
Source: JMIR Public Health Surveill. 2023 Mar 8;9:e45121. doi: 10.2196/45121 (PMC10034612; doi:10.2196/45121)

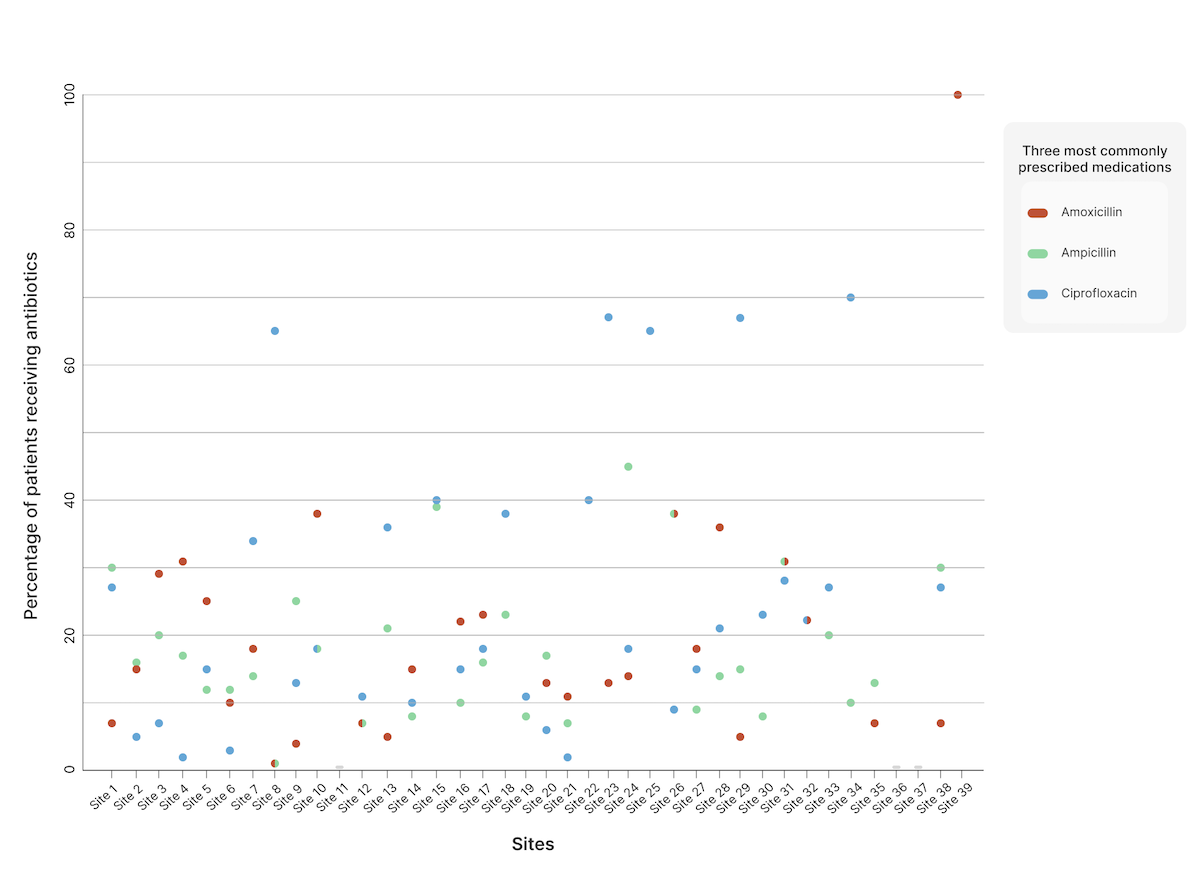

Supplement: Multimedia Appendix 1 [file publichealth_v9i1e45121_app1.png]

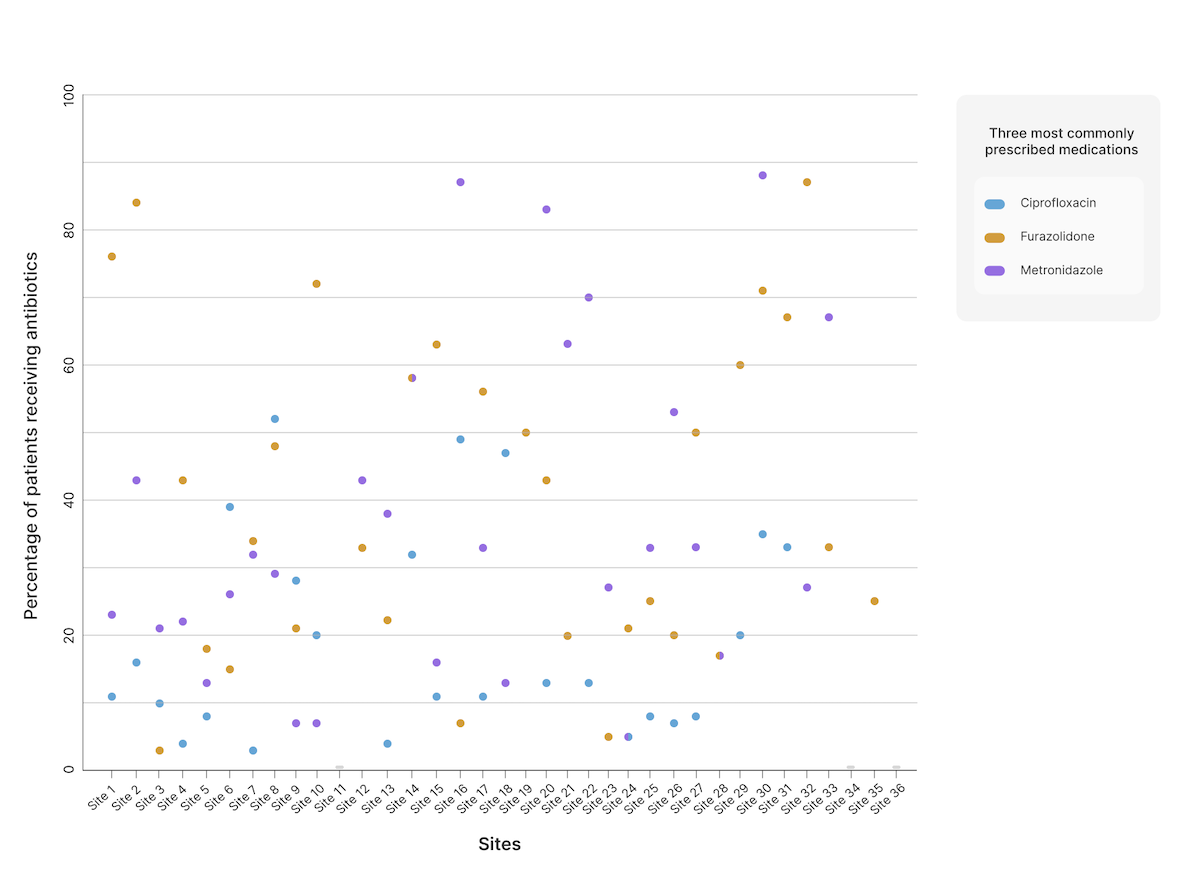

Supplement: Multimedia Appendix 2 [file publichealth_v9i1e45121_app2.png]
